# Undernutrition in obese older adults by fat percentage

**DOI:** 10.1007/s40520-023-02650-1

**Published:** 2024-01-23

**Authors:** Meris Esra Bozkurt, Tugba Erdogan, Nezahat Muge Catikkas, Serdar Ozkok, Cihan Kilic, Gulistan Bahat, Mehmet Akif Karan

**Affiliations:** 1 Division of Geriatrics, Department of Internal Medicine, Istanbul Medical School, Istanbul University Capa, 34390 Istanbul, Turkey; 2 Sancaktepe Training and Research Hospital, Sancaktepe, Istanbul, Turkey

**Keywords:** Undernutrition, Fatty obese, Older adults

## Abstract

**Objective:**

The prevalence of obesity by fat percentage has seen a steady increase in older adults in recent years, secondary to increases in fat mass in body composition, even in healthy aging. Malnutrition is a common geriatric syndrome with serious clinical outcomes. Increases in fat mass and waist circumference with healthy aging should not prevent the risk of malnutrition from being masked. Malnutrition is often ignored in obese older people due to low BMI cut-off values in many screening tests. The present study seeks to raise awareness of the need to assess the frequency of undernutrition and related factors in obese older adults.

**Methods:**

The data of 2013 community-dwelling patients aged ≥ 60 years who applied to a university geriatrics outpatient clinic between April 2012 and November 2022 were analyzed retrospectively, of which 296 were found to be obese based on fat percentage and were included in the study. Demographic data and the presence of any geriatric syndromes were obtained retrospectively from the patient files, functional status was assessed using the KATZ Activities of Daily Living (ADL) Scale and the LAWTON-BRODY Instrumental Activities of Daily Living Scale (IADL); frailty was screened using FRAIL-scale; and the sample was assessed for malnutrition using the Mini Nutritional Assessment-Short Form (MNA-SF), with undernutrition defined as an MNA-SF score of $\le 11.$ The patients’ fat percentage and weight were measured using a bioimpedance analyzer. Fatty obesity was defined using the Zoico methodology (fat percentage $\ge$ 27.3% for males, $\ge$ 40.7% for females)$,$ handgrip strength (HGS) was measured using a hand dynamometer, and probable sarcopenia was defined as low HGS based on regional cut-off values (35 kg for males, 20 kg for females).

**Results:**

The mean age of the 296 fatty obese older adults (102 males/194 females) was 74.4 + 6.5 years, and the median fat was 42.2% (27.4–59.5). Undernutrition was detected in 19.6% of the patients based on MNA-SF screening. A univariate analysis revealed age, sex, educational status, daily physical activity status, depression, difficulty in swallowing, chewing difficulty, probable sarcopenia, number of chronic diseases, and IADL to be associated with undernutrition, while a multivariate logistic regression analysis revealed depression [OR = 3.662, 95% CI (1.448–9.013), *p* = 0.005] and daily physical activity status [OR:0.601, 95% CI (0.417–0.867), *p* = 0.006] to be independently associated with malnutrition in obese older adults based on fat percentage.

**Conclusion:**

The present study clarifies the significance of undernutrition in obese older adults also in our country, and recommends undernutrition screening to be carried out, by fat percentage, on obese older adults, especially with depression and low daily physical activity.

## Introduction

In recent years, the prevalence of obesity has seen a marked increase in all age groups, including older adults [1–3]. In addition to the associated risks of cancer and cardiovascular disease, as common negative consequences of obesity, studies in literature have also reported obesity to be associated with impaired functionality and cognition in older adults [4–7]. Various changes occur in the body’s metabolism and composition with aging, the risk components of which increase the likelihood of obesity. Compensatory changes occur in body composition secondary to decreases in the metabolism rate and mobility, and these physiological changes, along with the aging process, lead to an increase in fat in the body composition and a decrease in muscle mass [8]. The most commonly used definition of obesity is the one put forward by the World Health Organization (WHO), which defines obesity as Body Mass Index (BMI) $\ge$ 30 kg/m^2^ [9]. The BMI definition cannot define the presence or absence of sarcopenia in the older adults defined as obese because it does not include the body’s muscle and fat mass. Sarcopenia is an important geriatric syndrome that is the result of malnutrition. The BMI definition ignores undernutrition and sarcopenia in older adults defined as obese for these reasons. However, the definition made according to BMI has revealed the necessity of new definitions for sarcopenic older adults, whose body fat percentage increases and muscle mass decreases with aging.

This has resulted in the inclusion in literature of obesity definitions based on body fat percentage [10, 11], including Zoico et al., who suggest that a fat percentile above the 60th percentile should be defined as obesity [10, 11]. The body fat percentage of older adults may vary due to regional differences. In a study of obesity in older adults in our region in recent years, obesity was defined as a body fat percentage of $\ge$ 40.7% in women and $\ge$ 27.3% in men based on the Zoico methodology, drawing attention to the need to define obesity in older adults with consideration of population-specific body fat percentages [11].

Malnutrition can have serious adverse clinical consequences for older adults [12–16], including frailty, sarcopenia, falls, hospitalization, loss of functionality and dependency [12–18].

Malnutrition is a significant geriatric syndrome due to its adverse outcomes and its bidirectional association with sarcopenia [17–19], and evaluating obesity based solely on BMI while neglecting the malnutrition risk in sarcopenic obese geriatric patients can have considerable consequences.

The present study investigates the prevalence of undernutrition and the associated factors in older adults with fatty obesity, defined based on the cut-off values specific to our region.

## Materials and method

### Study design and population

**Fig. 1:**
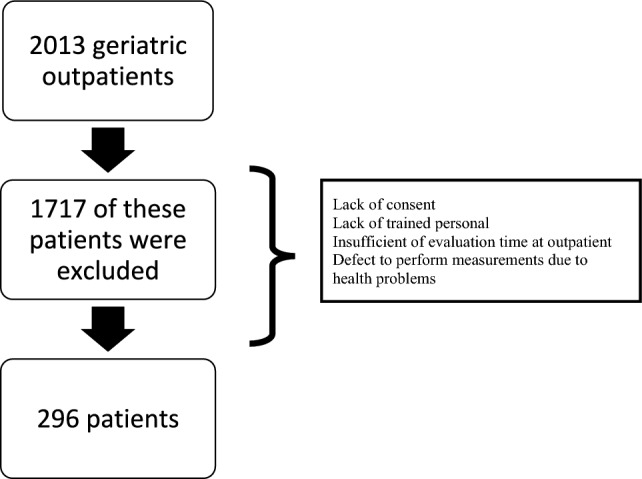
Flowchart of study participants

For this retrospective study, the data of 2013 community-dwelling patients aged ≥ 60 years who applied to a university geriatrics outpatient clinic between April 2012 and November 2022 were assessed, however, 1717 were subsequently excluded due to a lack of consent, a lack of trained personnel, insufficient evaluations at outpatient presentation and inability to undergo procedures due to health problems, including sequelae neurological deficit, neuropathy, pacemaker, severe osteoarthritis, severe hearing problems, advanced dementia and general conditional disorders. Patients aged ≥ 60 years who gave consent for their inclusion in the study and who were able to perform the measurement procedures were included in the study. The study population comprised 296 participants (Fig. 1), whose demographic data, including age, sex, geriatric syndromes, chronic diseases, and measurements, were obtained retrospectively from the patient files. Prior approval for the study was granted by the Local University Faculty of Medicine Clinical Research Ethics Committee (Reference number: 2023/222).

### Assessment

The participants’ age, sex, education status, marital status, smoking status, the number of diseases/drugs, and geriatric syndromes (difficulty in swallowing, chewing difficulty, falls, urinary incontinence, fecal incontinence, constipation, sleep disorders, chronic pain, frailty, probable sarcopenia, functional status and quality of life) were obtained from the patient files, as well as any chronic diseases**.**

Polypharmacy was defined as using ≥ 5 drugs [20] and the participants’ daily physical activity status was measured based on a multiple-choice questionnaire with the options: never, sometimes, 1–2 times per week and every day. The participants were questioned for any swallowing difficulties, chewing difficulties, falls, urinary incontinence, fecal incontinence, constipation, sleep problems and chronic pain. Chronic pain was assessed by asking the participants whether they had experienced pain for more than six months, and they were asked to provide a pain intensity score on a scale of 0–10 using the Visual Analog Scale (VAS) (0: least severe pain, 10: most severe pain in their life) [21]. Functional status was evaluated using the six-item KATZ Activities of Daily Living (ADL) Scale and the eight-item LAWTON-BRODY Instrumental Activities of Daily Living Scale (IADL) [22–24]. The participants scored 0 points for activities that could not be performed or that could be performed only with assistance, and 1 point for activities that could be performed alone. The ADL scale produces a total score in the range of 0 (dependent) to 6 (independent), while the IADL scale produces a total score in the range of 0 (dependent) to 8 (independent) [22–24]. Frailty was screened using the five-item FRAIL-scale, measuring fatigue, resistance, ambulation, illnesses and weight loss, in which a score of 0 indicated robust, scores of 1–2 indicated pre-frail and 3–5 indicated frail [25]. Quality of life was assessed using the European Quality-5 Dimension visual analog scale (EQ-5 VAS) [26], which is scored using a visual scale in which 0 denotes the worst health condition, and 100 the best health condition [26].

### Measurements

The height, calf circumference, mid-upper arm circumference, hip circumference, and waist circumference of the participants were measured using a regular stadiometer, and Body Mass Index (BMI) was calculated using the formula: body weight (kg)/height^2^ (m^2^) [9]. Handgrip strength (HGS) was measured using a Jamar hydraulic hand dynamometer. While seated in a standard backed chair, the respondent was asked to squeeze the dynamometer as hard as they could for 3 s in each hand with their arm next to their body, the elbow flexed 90°, and with the forearm and wrist in a neutral position, and the highest measured value was recorded [27]. Body composition measurements were made through a bioimpedance analysis (BIA, Tanita-BC532). After fat free mass measurements were achieved from the BIA device, skeletal muscle mass was computed with the equation below.

[Skeletal muscle mass = (Fat-free mass*0.566)] [10, 11, 28].

### Undernutrition definition

Malnutrition was screened using the Mini Nutritional Assessment-Short Form (MNA-SF), and undernutrition was defined as an MNA-SF score $\le 11 \left[29\right].$

### Obesity definition

Fat percentage and weight were measured using a BIA, and fatty obesity was defined based on the Zoico methodology (fat percentage $\ge$ 27.3% in males, 40.7% in females) [10, 11]$.$

### Probable sarcopenia definition

Probable sarcopenia was defined as low HGS based on regional cut-off values (35 kg for males, 20 kg for females) [30, 31]$.$

### Statistical analysis

The normality of continuous data was analyzed with a Kolmogorov–Smirnov test. For the descriptive statistics, continuous variables were expressed as mean ± standard deviation, median, and minimum–maximum values, while categorical variables were expressed as numbers (of subjects) and percentages. The differences between groups were determined with an independent samples *t* test, a Mann–Whitney *U* test and a Wilcoxon test. The Chi-square test and Fisher’s exact test for 2 × 2 probability tables suitable for categorical variables. A multivariate logistic regression analysis using the Enter method was used to determine the independent factors associated with undernutrition among the factors identified as significant in the univariate analyses. Multicollinearity was checked among the selected parameters, and Odds ratio (OR) and 95% confidence interval (CI) were used to express the associations. The fitness of the model was evaluated with a Hosmer–Lemeshow goodness-fit test. *p* values were based on two-sided tests and were accepted as statistically significant if < 0.05. The statistical evaluation of the study data was carried out using IBM SPSS Statistics (Version 20.0. Armonk, NY: IBM Corp.)

## Results

Included in the study were 296 fatty obese older adults (102 male/194 female) [mean age:74.4 + 6.5 years (61–92)], among which 19.6% were undernourished (Fig. 1). The median BMI of the study group was 32.8 (21.7–58.7) kg/m^2^ (Table 1).

**Table 1:** Characteristic data of the study population by nutritional status

	Undernutrition (*n* = 58)	Normal nutrition (*n* = 238)	Total (*n* = 296)	*p* value
	19.60%	80.40%	100%	N/A
Age	76 + 6.5 (61–87)	74 + 6.5 (61–92)	74.4 + 6.5 (61–92)	**0.037**^Θ^
Gender (*n*, %)				
Male	13 (22.4%)	89 (37.4%)	102 (34.5%)	**0.031**^Θ^
Female	45 (77.6%)	149 (62.6%)	194 (65.5%)	
Education (*n*, %)				
Illiterate	17 (29.3%)	26 (10.9%)	43 (14.5%)	
Literate	9 (15.5%)	38 (16%)	47 (15.9%)	
Primary school	16 (27.6%)	91 (38.2%)	107 (36.1%)	**0.027**^Θ^
Middle school	4 (6.9%)	25 (10.5%)	29 (9.8%)	
High school	7 (12.1%)	27 (11.3%)	34 (11.5%)	
College-faculty	5 (8.6%)	27 (11.3%)	32 (10.8%)	
Master-doctorate	0 (0%)	4 (1.7%)	4 (1.4%)	
Marital status (*n*, %)				
Single	0 (0%)	1 (0.4%)	1 (0.3%)	
Married	28 (48.3%)	137 (57.6%)	165 (55.7%)	0.573
Divorced	1 (1.7%)	4 (1.7%)	5 (1.7%)	
Widowed	29 (50%)	96 (40.3%)	125 (42.2%)	
Smoking status (*n*, %)				
Smoker	12 (5%)	7 (12.1%)	19 (6.4%)	0.142
Quit	62 (26.1%)	15 (25.9%)	77 (26%)	
Never	164 (68.9%)	36 (62.1%)	200 (67.6%)	
Daily physical activity status (*n*, %)				
Never	26 (44.8%)	38 (16%)	64 (21.6%)	
Sometimes	3 (5.2%)	5 (2.1%)	8 (2.7%)	** < 0.001**^Θ^
1–2 times per week	19 (32.8%)	84 (35.3%)	103 (34.8%)	
Everyday	10 (17.2%)	111 (46.6%)	121 (40.9%)	
Chronic disease (*n*, %)				
Dementia	7 (12.1%)	19 (8%)	26 (8.8%)	0.324
Depression	16 (27.6%)	25 (10.5%)	41(13.9%)	**0.001**^Θ^
Diabetes mellitus	27 (46.6%)	93 (39.1%)	120 (40.5%)	0.298
Rheumatological diseases	1 (1.7%)	4 (1.7%)	5 (1.7%)	0.982
Congestive heart failure	3 (5.2%)	17 (7.1%)	20 (6.8%)	0.592
Chronic kidney disease	7 (12.1%)	10 (4.2%)	17 (5.7%)	**0.021**^Θ^
COPD	6 (10.3%)	16 (6.7%)	22 (7.4%)	0.346
Geriatric syndromes (*n*, %)				
Difficulty in swallowing^⊥^	9 (17.6%)	13 (6.9%)	22 (8.5%)	**0.009**^Θ^
Chewing difficulty^X^	17 (33.3%)	29 (14.4%)	46 (18.3%)	**0.002**^Θ^
Falls	27 (46.6%)	90 (37.8%)	117 (39.5%)	0.222
Urinary incontinence	37 (63.8%)	104 (43.7%)	141 (47.6%)	**0.006**^Θ^
Fecal incontinence	5 (8.6%)	15 (6.3%)	20 (6.8%)	0.528
Constipation	25 (43.1%)	58 (24.4%)	83 (28%)	**0.004**^Θ^
Sleep disorders	31 (53.4%)	115 (48.3%)	146 (49.3%)	0.585
Chronic pain	37 (63.8%)	140 (58.8%)	177 (59.8%)	0.489
Frailty	55 (94.8%)	119 (50%)	174 (58.8%)	** < 0.001**^Θ^
Probable sarcopenia (27/16 kg)	8 (13.1%)	17 (7.1%)	25 (8.4%)	0.102
Probable sarcopenia(35/20 kg)	28 (48.3%)	76 (31.9%)	104 (35.1%)	**0.019**^Θ^
Quality of life (0–100)*	60 (10–100)	70 (10–100)	70 (10–100)	0.324
Polypharmacy (*n*, %)	42(72.4%)	152 (63.9%)	194 (65.5%)	0.219
Number of chronic drugs*	7 (0–15)	6 (0–16)	6 (0–16)	**0.026**^Θ^
Number of chronic diseases*	4 (0–10)	3 (0–9)	4 (0–10)	**0.001**^Θ^
ADL*	5 (0–6)	6 (1–6)	6 (0–6)	** < 0.001**^Θ^
IADL*	7 (0–8)	8 (0–8)	8 (0–8)	** < 0.001**^Θ^

Data are given as mean ± standard deviation, median (interquartile range) or number (%) as applicable

*ADL* Activities of daily living, *IADL* Instrumental activities of daily living, *COPD* Chronic obstructive pulmonary disease

*Given data as median (interquartile range)

^⊥^Difficulty in swallowing data was present in 258 participants

^Χ^Chewing difficulty data was present in 252 participants

^Θ^Significant *p* values

Univariate analyses revealed a statistically significant association between undernutrition and age (*p* = 0.037), sex (*p* = 0.031), education status (*p* = 0.027), daily physical activity (*p* < 0.001), depression (*p* = 0.001), chronic kidney disease (*p* = 0.021), difficulty in swallowing (*p* = 0.009), chewing difficulty (*p* = 0.002), urinary incontinence (*p* = 0.006), constipation (*p* = 0.004), frailty (*p* < 0.001), probable sarcopenia (35/20 kg) (*p* < 0.019), number of diseases (*p* = 0.026), number of drugs (*p* = 0.001), ADL (*p* < 0.001)**,** IADL (*p* < 0.001) (Table 1).

Univariate analyses revealed a statistically significant association between undernutrition and hand grip strength (*p* = 0.001), fat free mass (*p* < 0.001), skeletal muscle mass (*p* < 0.001), calf circumference (*p* = 0.034) and mid-upper arm circumference (*p* = 0.038). However, no statistically significant relationship was found between BMI (*p* = 0.282) and undernutrition (Table 2).

**Table 2:** Characteristic measurements data by nutritional status of the study population

	Undernutrition (*n* = 58)	Normal nutrition (*n* = 238)	Total (*n* = 296)	*p* value
	19.6%	80.4%	100%	N/A
Hand grip strength (kg)*	22 (10–46)	26 (8–58)	24 (8–58)	**0.001**^**Θ**^
Fatty percentage (%)*	42.7 (27.9–49.6)	42.2 (27.4–59.5)	42.2 (27.4–59.5)	0.181
Fat-free mass (kg)*	42.2 (31–71.6)	45.8 (31.7–72.1)	45.2 (31–72.1)	** < 0.001**^**Θ**^
Skeletal muscle mass (kg)*	23.9 (17.5–40.5)	25.9 (17.9–40.8)	25.6 (17.5–40.8)	** < 0.001**^**Θ**^
Calf circumference (cm)*	38 (30–45)	39 (29–50)	39 (29–50)	**0.034**^**Θ**^
Mid-upper arm circumference (cm)*	30 (23–37)	30 (25–44)	30 (23–44)	**0.038**^**Θ**^
Hip circumference (cm)*	114 (95–114)	113 (94–163)	113 (94–163)	0.990
Waist circumference (cm)*	106(92–123)	109 (88–142)	109 (88–142)	0.085
BMI (kg/m^2^)*	32.4 (23.5–52)	32.8 (21.7–58.7)	32.8 (21.7–58.7)	0.282

*BMI* Body Mass Index

*Given data as median (interquartile range)

^Θ^Significant *p* values

Undernutrition was found to be statistically related with fat free mass (*p* < 0.001), skeletal muscle mass (*p* < 0.001). Also, statistically significant relationship was found between fat free mass (*p* < 0.001) and skeletal muscle mass (*p* < 0.001) with the hand grip strength.

In multivariate analyses including factors independently associated with undernutrition in fatty obese older adults were adjusted for age, gender, education status, daily physical activity status, chronic kidney disease, depression, difficulty in swallowing, chewing difficulty, probable sarcopenia (35/20 kg), number of chronic diseases, IADL. After adjustment independently associated factors found with undernutrition the presence of depression (OR 3.662 *p* = 0.005), and daily physical activity status (OR 0.601, *p* = 0.006) in multivariate analyses (Table 3).

**Table 3:** Results of multivariate regression analysis: factors independently associated with undernutrition in fatty obese older adults after adjustment for age, gender, education status, daily physical activity status, chronic kidney disease, depression, difficulty in swallowing, chewing difficulty, probable sarcopenia(35/20kg), number of chronic diseases, IADL

	*p*	OR	95%Cl	
			Lower	Upper
Age	**0.642**	**0.986**	**0.927**	**1.048**
Gender	**0.693**	**1.218**	**0.458**	**3.241**
Education status	**0.979**	**1.003**	**0.775**	**1.299**
Daily physical activity status	**0.006**^**Θ**^	**0.601**	**0.417**	**0.867**
Chronic disease				
Chronic kidney disease	**0.126**	**2.840**	**0.747**	**10.801**
Depression	**0.005**^**Θ**^	**3.662**	**1.448**	**9.013**
Difficulty in swallowing	0.972	1.021	0.329	3.171
Chewing difficulty	0.205	1.716	0.745	3.952
Probable sarcopenia (35/20 kg)	0.983	1.014	0.287	3.579
Number of chronic diseases	0.685	1.022	0.920	1.136
IADL	0.053	0.839	0.702	1.003

*CI* Confidence interval, *IADL* instrumental activities of daily living, *OR* Odds ratio

^Θ^Significant *p* values

## Discussion

The results of the present study of 296 older adults identified as obese based on their fat percentage revealed a risk of malnutrition in 19.6%, and univariate analyses identified many factors (age, sex, education status, daily physical activity, chronic kidney disease, depression, difficulty in swallowing, chewing difficulty, urinary incontinence, constipation, frailty, probable sarcopenia (35/20 kg), number of diseases, number of drugs, ADL, IADL, calf circumference, mid-upper arm circumference) that were independently associated with malnutrition risk.

In the multivariate analyses, presence of depression and daily physical activity status were identified as being associated with malnutrition risk in older obese adults. The results of the present study identified malnutrition in 19.6% of the obese older adult respondents based on fat percentage. The most common definition of obesity in older adults and in all societies is based on BMI, as recommended by a WHO Expert Committee [9], and most studies of older adults are based on this definition. A review of literature reveals malnutrition risk in obese older adults is most commonly based on the BMI definition, and that the prevalence of malnutrition varies from one society to another [32–36]. According to the findings of one study, even in Central Africa, where the risk of malnutrition is high, the prevalence of obesity among older adults is 8.8% [32]. In Turconi et al.’s study of 184 older adults over the age of 60 years in Italy, 39.1% were found to be obese based on the BMI definition, although most of the participants had a low socioeconomic level [33], and 12% of the older participants in the study were found to be malnourished according to MNA-Long Form [33]. In Kaiser et al.’s validation study for the Mini Nutritional Test Short Form (MNA-SF), the risk of malnutrition was ignored in 6% of the 656 participants when the risk of malnutrition was evaluated based on BMI [34].

In Bahat et al.’s study of nursing home patients, the prevalence of nursing home residents with a BMI ≥ 25 was found to be 56.6%, and of these overweight + obese adults, 17.3% were found to be at risk of undernutrition. In addition, the prevalence of obesity among older adults living in nursing homes was 21.6%, and 9.1% were found to be at risk of undernutrition [35]. Soysal et al.’s cross-sectional retrospective studies of 1911 older adults revealed a prevalence of obesity of 48.7%, and 32.3% who were at risk of undernutrition [36].

In their study, Özkaya et al. found that 187 of the 596 older adult participants in their study under 1-year follow-up in the outpatient clinic were overweight + obese, and 49.7% were at undernutrition risk [37]. Our results differ significantly from those of other studies, which may be attributed to differences in the definition of obesity. The definition of obesity recommended by WHO, which is in common use around the world, ignores the changing body composition of older adults, and so obesity in the present study was defined using the Zoico methodology, which takes into account the changes in body composition with aging, and has been adopted in other studies to date [10, 11, 38, 39].

In the first of these studies, 167 community-dwelling women aged 67–78 were compared with 120 premenopausal Italian women between the ages of 20–50, and those with a fat percentile above the 60th percentile were identified as obese [10].

In another study conducted in France involving 1,308 healthy women aged 75 and over, those with a fat percentile over the 60th percentile were defined as obese. When they defined obesity, this as having a body fat percentage above 40%, around 36% of the participants were found to be obese based on their body fat percentage [38]. A study by Kim et al. in Korea involving 526 participants, obesity was defined as above the 60th percentile of fat percentage measured by Dual-Energy X-Ray Absorptiometry (DXA), and defined as body fat percentages above 20.21% and 31.71% for males and females, respectively. The authors reported a prevalence of obesity of around 15–20% based on body fat percentage in participants over the age of 60 years [39]. In a study by Lii et al. in China involving 2393 older adults, obesity was found as fat percentage ≥ 25% in males and ≥ 35% in females when obesity was defined as the 60th percentile of older adults’ fat percentage. [40]. In this study, when obesity was defined as above the 60th percentile in fat percentage, the prevalence of obesity in older adults was found to be 41.8% [40]. In Turkey, Bahat et al., when defining obesity as a fat percentile above the 60th percentile, reported around 40% of older adults to be obese [13]. In the present study, daily physical activity status and the presence of depression, which is a chronic disease, were found to be associated with a risk of malnutrition in fatty obese older adults similar to the cited studies. [10, 11, 38, 39]. These important studies, however, have not been followed up by further studies investigating the frequency of malnutrition risk and the related factors in fatty obese older adults.

The strengths of our study include the wealth of data it provides on a large number of older community-dwelling adults, and its status as the first study to examine undernutrition screening in obese adults based on fat percentage in our community. The most significant strength of the present study is its use of a definition of obesity based on the physiological body composition in older adults. The limitations of the present study, on the other hand, include the unavailability of some data due to the retrospective study design, and the exclusion of people at serious risk of malnutrition, such as those with heart failure, due to the use of BIA. Another limitation is related to the lack of awareness among physicians about the term “obesity” when based on fat percentage, leading to a lack of studies in literature for comparison.

## Conclusion

We confirm that also in our country, as found in previous studies [36–40], the prevalence of undernutrition in fatty obese older adults is high. We identified depression and low daily physical activity as related factors in undernutrition in fatty obese older adults. Although our study is important in terms of the presented results, prospective studies are needed to allow a more detailed comparison of the results.

## Data Availability

The data is available from the authors upon reasonable request.

## Code availability

N/A.
